# Assessing Bradykinesia in Parkinsonian Disorders

**DOI:** 10.3389/fneur.2013.00054

**Published:** 2013-06-03

**Authors:** Gian Pal, Christopher G. Goetz

**Affiliations:** ^1^Department of Neurological Sciences, Rush University Medical CenterChicago, IL, USA

**Keywords:** bradykinesia, UPDRS, MDS-UPDRS, technology, Parkinson disease, PSP

## Abstract

**Objective:** Bradykinesia is one of the clinical hallmarks of Parkinson’s disease (PD) and atypical Parkinsonian syndromes. Clinical ratings scales and technology-based assessments have been developed to measure bradykinesia. We review the different tools that exist for measurement of bradykinesia and analyze their reliability and applicability to PD and atypical Parkinsonian syndromes.

**Methods:** We summarize data on the factor structure of the two primary scales used to assess PD, the Unified Parkinson’s Disease Rating Scale (UPDRS) and Movement Disorder Society revision of the UPDRS, the MDS-UPDRS. We review how these scales have been used in atypical Parkinsonian syndromes, specifically Progressive Supranuclear Palsy (PSP). Finally, we report on the different technology-based tools being used to assess bradykinesia.

**Results:** The UPDRS is a useful measure of PD function and disability with six clinically distinct factors, three of which pertain to bradykinesia. The MDS-UPDRS has shown high internal consistency and correlation with the original UPDRS. Factor analysis of the UPDRS in PSP reveals five clinically distinct factors, two of which are independent bradykinesia factors. Thus the UPDRS and MDS-UPDRS are reliable and applicable scales for PD and the UPDRS can be used to assess bradykinesia in PSP. Technology-based tools for measuring bradykinesia include gyrosensors, Coordination Ability Test System, Brain Test, quantitative digitography, Motus motion analysis system, precision real-time image-based motion analysis, and the At-Home Testing Device. These tools have been compared to the UPDRS motor subscale and are effective in assessing bradykinesia.

**Conclusion:** The UPDRS and MDS-UPDRS are well-established measures of bradykinesia that are applicable and useful in PD. The UPDRS is also been shown to be applicable to PSP. Different technologies exist to measure bradykinesia, though further work is needed to validate these assessment tools and bring them to clinical practice.

## Introduction

Bradykinesia is one of the clinical hallmarks of Parkinson’s disease (PD) and atypical Parkinsonian syndromes and can be measured with clinical tools in the form of rating scales, as well as technology-based assessments. These different tools are complementary and can be utilized separately or together, depending on the assessment goals.

## Clinical Rating Scales

Clinical rating scales are instruments that provide a numeric value to clinical signs considered pertinent to the assessment of a given condition. In PD, a number of scales were developed prior to 1980, including the Columbia University Rating Scale (CURS), the Webster Scale, and the Parkinson’s Disease Impairment Scale (PDIS) (Ramaker et al., 2002). These scales evaluated bradykinesia, combining slowness, hesitation, breakdown of smooth movement, and amplitude decrements into a combined assessment that was ranked from normal (0) to the highest allowable score for severe bradykinesia, meaning unable or barely able to execute a given motor task. To cull the most important features of PD from these different scales, an effort to develop scales using the designation “Unified” evolved with two key scales, the Unified Parkinson’s Disease Rating Scale (UPDRS) and its recent update, the Movement Disorder Society revision of the UPDRS (MDS-UPDRS). An additional scale, the Modified Bradykinesia Rating Scale for PD (Heldman et al., 2011) has also been used in clinical practice, though the gold standard for assessment of bradykinesia remain the UPDRS and the MDS-UPDRS.

### The unified Parkinson’s disease rating scale

The UPDRS was developed to assess PD function, and bradykinesia is rated by observation of several tasks, each scored using five options from normal (0) to severe impairment (4). Eleven of the total 27 objective ratings measure bradykinesia through finger taps, hand movements, rapid alternating movements of the hands, leg agility, arising from a chair, gait, and body bradykinesia and hypokinesia. The factor structure and internal consistency has been studied in 294 PD patients in the “on” state, that is during the period of the day when they experience positive dopaminergic medication effects. Factor structure analysis was performed and six factors were obtained which accounted for approximately 78% of the sample variance. Three of these six factors concerned bradykinesia. Items assessing axial function, balance, and gait clustered together into one factor. Two additional distinct factors, right side extremity and left side extremity bradykinesia were also identified. There was no correlation between bradykinesia testing on the right and left sides. These UPDRS measures of bradykinesia correlated well with other measures of PD disability, i.e., Hoehn and Yahr (HY) staging and Schwab and England ADL scale (SE) (Stebbins and Goetz, 1998).

In order to confirm that the statistic stability of the UPDRS factor structure across different groups of patients with varying levels of PD impairment, a subsequent analysis was performed with PD patients in the off state, that is when medication effects were not apparent. In a sample of 200 patients examined in the off state, with identical analytic methods used in the prior publication, six factors were again identified and accounted for approximately 70% of the variance, three of which related to bradykinesia. All assessments of axial function, speech, facial expression, balance, and gait clustered together into one factor. Again, two additional distinct factors assessing bradykinesia affecting the extremities were identified, right and left. There was no correlation between bradykinesia testing on the right and left sides. There was a significant relationship between the individual factors assessing bradykinesia from the UPDRS and the HY stage. The results were highly consistent between the two studies, confirming the reliability and validity of the UPDRS as a scale for assessment of bradykinesia in PD both when patients are experiencing medication benefit and when they are not (Stebbins et al., 1999).

The UPDRS has also been applied to the evaluation of parkinsonian syndromes outside of PD. Progressive Supranuclear Palsy (PSP) shares some clinical features with PD and is the most common and best recognized condition among the atypical parkinsonian syndromes. A study analyzing the factor structure of UPDRS Motor Examination in 175 PSP patients revealed five factors which accounted for approximately 64% of the sample variance, two of which assess bradykinesia. Items assessing axial bradykinesia and gait clustered into one factor, and items assessing bradykinesia of the extremities, right and left combined, clustered into a separate factor. There was no correlation between these two factors, and each of the factors assessing bradykinesia was significantly related to HY stage. No side-to-side difference in bradykinesia was found in the factor analysis for PSP patients, which follows the typical clinical presentation of PSP as a symmetrical illness in contrast with the asymmetry of typical PD. These results support the validity and reliability for the use of the motor scale of the UPDRS in measuring bradykinesia for patients with PSP (Cubo et al., 2000).

### The movement disorder society-sponsored revision of the unified Parkinson’s disease rating scale

The MDS-UPDRS is a revision of the original UPDRS, aimed at retaining the strengths of the original scale and resolving some of the ambiguities and identified weaknesses. In regards to bradykinesia, the main addition to the MDS-UPDRS involves toe tapping. Conceptually, the UPDRS and the MDS-UPDRS are not identical, as the new scale was designed specifically to detect very mild changes not captured in the original scale. As such, the five options for each item are “normal,” “slight,” “mild,” “moderate,” and “severe” impairment whereas the original scale focused more on advanced disease. Thus for most items, and specifically the bradykinesia items, direct item-to-item mapping is not possible between the two scales. The factor structure of the MDS-UPDRS was evaluated in a study of 877 native English-speaking PD patients of diverse race/ethnicity representations. Exploratory and confirmatory analysis of the individual parts of the MDS-UPDRS identified a factor structure that was statistically consistent and clinically meaningful for all parts. Eigenvalues and scree plots informed the exploratory factor analysis which determined the number of factors that best represented the data. Confirmatory analysis was used to assess dimensionality, with a comparative fit index (CFI) greater than or equal to 0.90 as an acceptable fit. For the Motor Examination the CFI = 0.91, and seven factors were identified, four of which pertained to bradykinesia (midline function, bradykinesia right upper extremity, bradykinesia left upper extremity, lower limb bradykinesia, including both right and left legs). Further, there was a strong concurrent validity based on high correlations between the MDS-UPDRS and the UPDRS Part III (*r* = 0.96). These results support the reliability and validity of the MDS-UPDRS in PD patients. This has led to an increasing trend in the field to adopt this newer scale as the “gold standard” (Goetz et al., 2008).

## Technology-Based Tools

Although clinical rating scale scores are based on objective face-to-face examination of a patient, clinical judgment, and some degree of subjectivity are involved. Further, the limited item scoring options do not provide a wide array of choices with continuous variables. To help quantify bradykinesia more objectively, a variety of different technology-based tools have been developed. These technology-based tools were correlated with clinical examination scores from the UPDRS and/or the MDS-UPDRS to assess validity and reliability, since clinical scales are the gold standard for most researchers and regulatory agencies. We performed a search of PubMed.gov and included articles published since the year 2000. Specific tools where the authors made a correlation between the standard clinical examination tools, (i.e., elements of the UPDRS and/or the MDS-UPDRS) and the technology-based assessments are discussed below.

### Gyrosensors

The gyrosensor measures angular movement in terms of angular velocity and limits gravitational artifact. This sensor was used to assess bradykinesia during finger taps in 40 patients with PD and 14 age-matched control subjects. There were four performance indices that were derived from the sensor signal – root-mean-squared (RMS) angular velocity, RMS angle, peak power, and total power. RMS velocity and RMS angle were expected to represent slowing of finger-tapping motion and reduction in amplitude due to bradykinesia, respectively. Peak power and total power were expected to represent the intensity of the main movement component of finger tapping and the total intensity of movement. There was moderate correlation (*r* = 0.73–0.80, *P* < 0.001) between these performance indices and the clinical finger tap score. Each of the different performance indices was able to differentiate patients from controls (*P* < 0.001). Cronbach’s alpha was 0.85 and the intraclass coefficient was 0.74, showing a high inter-rater reliability of the FT score (Kim et al., 2011).

### Brain test

A computer software program (BRAIN TEST©), which is also based on the finger-tapping test, has been used to assess bradykinesia. Subjects are instructed to tap marked standardized keyboard keys successively for 60 s with their index finger as fast and as accurately as possible. A Kinesia score (KS) is obtained which corresponds with the total number of keystrokes per 60 s, is used as a measure of bradykinesia. To exclude inter-rater variability, the same neurologist graded all cases. In 154 PD patients, there was a significant correlation between KS and the UPDRS Part III (*r* = −0.600, *P* < 0.001). Seventy-three PD patients were also compared with age-matched control subjects and the PD patients different significantly with regard to KS (70.3 ± 27.3 keystrokes/min vs. 126.0 ± 21.7 keystrokes/min, *t* = 13.6; *P* < 0.001). Based on KS and other measures, correct classification into the control or Parkinson group was achieved in 85.6% of subjects, showing this is a valid measure of bradykinesia (Homann et al., 2000).

### Coordination ability test system

Another tool that has been used to quantify bradykinesia is the Coordination Ability Test System (CATSYS). In a study of 44 patients with PD, this device was used to assess bradykinesia, specifically measuring tasks assessed in the UPDRS, pronation/supination and finger tapping using a touch sensitive recording plate. Using this system, bradykinesia was assessed and measures were compared to corresponding UPDRS items. There was a significant correlation of the mean value for CATSYS pronation/supination with the corresponding UPDRS item (ρ = −0.411, *P* < 0.014), but there was no relationship with CATSYS finger-tapping values with the corresponding UPDRS bradykinesia items (*P* > 0.05). Though the CATSYS was reliable in discriminating between PD and control subjects, it did not clearly show validity compared to the UPDRS (Papapetropoulos et al., 2010).

### Quantitative digitography

A musical instrument digital interface (MIDI) keyboard has been used to assess bradykinesia in a technique termed quantitative digitography (QDG). For each finger, the means of key-strike velocity, duration of finger strike, and the interval between strikes were calculated during a repetitive alternating finger-tapping task (RAFT) over 30 s. In 33 patients who were off PD medications, QDG scores were correlated with the UPDRS part III scores, specifically with the CV duration of finger strike (*r* = 0.66; *P* < 0.001), key-strike velocity (*r* = −0.61; *P* < 0.001), and the CV of the interval between strikes (*r* = 0.56; *P* < 0.001). A model combining three QDG variables, key-strike velocity (Vel), interval between strikes (Int), and the CV of the duration of the finger strike (CVDur) together best predicted the UPDRS III scores (*r* = 0.704; *P* = 0.001). QDG was compared to the UPDRS III to assess effects of therapy (medication and STN DBS) on motor disability. Both the UPDRS and QDG measurements are sensitive to improvements of overall and fine motor control from medication and DBS, indicating its validity and reliability. Interestingly, QDG was able determine a significant difference in the effect of the two different therapies (medication vs. DBS), that was not detectable using the UPDRS (Taylor Tavares et al., 2005).

### Motus motion analysis system

Technology-based tools have been used in other aspects of the surgical arena. Anecdotal reports suggested that patients’ motor symptoms improve immediately following the STN DBS surgical procedure prior to pulse generator activation, a phenomenon termed the “microlesion” effect. A quantitative measure of bradykinesia, root mean square velocity of angular movement (Vrms), was used to formally study this concept during a quantitative repetitive wrist pronation-supination (qrWPS) task using the Motus motion analysis system in 101 patients. Measures of bradykinesia were collected at three time points during the STN DBS procedure: pre-MER (microelectrode recording), post-MER, and during high-frequency electrical stimulation from the quadripolar electrode. Patients were assessed preoperatively in the off medication condition and the Vrms value showed an inverse correlation with a UDPRS Motor Examination bradykinesia subscore (Spearman rank correlation, *r* = −0.54; *P* = 0.001; *n* = 61). Higher angular velocity indicated less limb bradykinesia and validated Vrms as a measure of bradykinesia. Using this technology, patients Vrms scores improved by 28.0% (*P* < 0.003) between pre- and post-MER, and 41.0% (*P* < 0.003) between post-MER and intraoperative DBS (Koop et al., 2006).

### Precision real-time image-based motion analysis

Variations of the finger-tapping movement have also been studied using technology-based tools using wire free contact sensors. This technology, called Precision Real-time Image-based Motion Analysis (PRIMAS) has been used to develop a novel parameter, a finger-tapping test score (FTTS), to rate the finger-tapping movement. The FTTS for each Parkinsonian patient was found to be smaller than the average for healthy subjects. This was also true for Parkinsonian patients who were HY stage I, illustrating that this technology could be used to distinguish patients with early disease from healthy subjects. However, the reliability of the test among subjects was variable, and improved with repeated testing, indicating the presence of a learning effect. Also, though finger tapping is a part of the motor subscore of the UPDRS, the investigators did not evaluate the validity of this tool compared to the actual UPDRS scale (Jobbágy et al., 2005).

### At-home testing device

Since most long-term clinical trials assess the UPDRS scores at baseline and at fixed time intervals, a tool was developed which would measure changes more frequently and at home, termed the At-Home Testing Device (AHTD). To assess bradykinesia, patients performed a series of motor tasks using a computer module and the data obtained could be electronically transmitted to a central computer bank, which was found to be technically reliable. In a study of 50 untreated PD patients with less than 5 years of disease, the mean monthly change of the AHTD data was compared with changes in office-based UPDRS scores at 3 and 6 months. The AHTD measured bradykinesia through alternating finger tapping (digitography) and hand tapping. Over the 6-month period, patients declined in overall UPDRS motor function (*P* = 0.009) but measures of AHTD bradykinesia did not detect changes earlier or more robustly than the clinical scales, indicating this is not a valid tool for assessment of bradykinesia (Goetz et al., 2009).

## Discussion

Bradykinesia, one of the hallmarks of PD is effectively measured by the UPDRS and the MDS-UPDRS, and the clinical exam remains the gold standard for evaluation of Parkinsonian patients. These scales have also been effectively applied to atypical Parkinsonian syndromes such as PSP. Additional scales such as the Modified Bradykinesia Rating Scale have also been developed and are being used in both in patient and clinical trials. However, the UPDRS and the MDS-UPDRS remain the most widely accepted scales. Technology-based tools are aimed at quantifying bradykinesia objectively to increase diagnostic accuracy and to help monitor subtle clinical changes when therapies are initiated. To date, no new treatment has received regulatory approval based on use of technology-based objective measures of bradykinesia or any other objective measure of parkinsonism.

There remains a role and need for technology-based tools, and this need may grow with further research. Although both the UPDRS and MDS-UPDRS have good inter-rater reliability, some variation exists among different raters evaluating the same subject. Technology-based tools such as the gyrosensor have the potential to reduce the inter-rater variability of clinical scales. Also, technology-based tools may be useful in measuring the effects of therapies on motor function that may be subtle in early stages of PD as was shown with QDG and PRIMAS. The UPDRS and MDS-UPDRS may assign the same summary score to patients with different aspects of impairment. For example, a finger-tapping score is based on of “2” is given to three possible attributes: slowness, fatiguing, and arrested movement. A patient who can maintain the rhythm of the task but does the movements slowly is given the same score as a patient who can perform the repetitions faster, but with freezes or hesitations in movement. Quantitative analysis with tools such as QDG may help parse out these differences, and may be a more sensitive method of assessing a patient’s unique motor profile so that it can be accurately tracked over time (Bronte-Stewart et al., 2000). Tools such as Motus and QDG are already being used in surgical evaluation of DBS patients where quantitative measures of bradykinesia help to shed light on the pathophysiology and effects of DBS that are not discerned by a clinical rating scale. However, there are also limitations to certain tools, as noted with Brain Test©. Also, the availability of the technology-based tools as discussed above is currently limited, although greater commercial and research-based availability can be envisioned for the future.

The practicality of technology-based rating measures still needs to be established. Though bulky and expensive equipment will never be widely adopted, the new availability of I-Pads and I-Phones suggests that easy to use measures might allow very frequent and home-based documentation more linked to daily living than the forced protocols of office examinations. Technology-based tools need to be validated with larger patient populations crossing the gamut of parkinsonian disabilities. Further, normative data on the tool that is being developed is critical so that the patient population with disease can be appropriately assessed. The tool being assessed should also be studied over an appropriate time span. The UPDRS and MDS-UPDRS are typically assessed at 3- or 6-month intervals in clinical trials and these scales are sensitive enough to detect changes in motor decline within this time period. In contrast, the ATHD was not able to detect clinical worsening of bradykinesia that was evident on the UPDRS. Thus the tool being developed must be valid not only in discriminating between patients with disease compared to control subjects, but must also be able to detect changes over standard intervals of time. Also, we are already seeing improvements from a technological perspective. Heldman et al. (2011) used motion sensors to assess bradykinesia to measure the reliability of the Modified Bradykinesia Rating Scale and compared it with the UPDRS. They were able to find that the MBRS was reliable as compared to the UPDRS, and were also able to correlate the MBRS data with the kinematic measures obtained from the motion sensors. Mera et al. (2012) have taken this a step further and developed a home-based automated method based on these motion sensors that can be used for assessment of PD that measures both tremor and bradykinesia. The disease progression as measured by clinical tools is often non-linear, whereas the amount of data generated by technology-based tools may be sensitive enough to capture the slowly progressive decline in Parkinsonian disorders. However, the decline in PD is not necessarily linear and certainly can be variable from patient to patient, so such technology-based tools may be helpful in assessing the trajectory of disease progression in different patients. The prospects for increasingly simple and valid measurement tools are high.

Additional cautionary caveats need to be emphasized as these tools are developed. First, the numbers generated must have a direct bearing on the clinical state under question, because an improvement in a movement variable, even if statistically significant, has little importance if it does not correlate strongly with clinically appreciated improvements. Further, such tools have the potential to register almost limitless information and over-sampling can lead to data sets difficult to interpret. As in the case of the BRAIN TEST© software, the learning effects need to be considered, as well as confounding influences of cognitive function, motivation, tremor, and the topographic distribution of the motor deficit being measured. As such, rating scales and technology-based tools s are likely to be used in research and clinical care as complementary strategies for tracking disease progression and response to treatment. Private technology firms, pharmacologic industry, PD foundations, and governments are increasingly vested in accurate measurement of PD, so it is likely that rapid advances in bradykinesia monitoring will be achieved.

## Conflict of Interest Statement

The authors declare that the research was conducted in the absence of any commercial or financial relationships that could be construed as a potential conflict of interest.
